# Differences in Brain Metabolic Impairment between Chronic Mild/Moderate TBI Patients with and without Visible Brain Lesions Based on MRI

**DOI:** 10.1155/2016/3794029

**Published:** 2016-07-26

**Authors:** Keiichi Ito, Yoshitaka Asano, Yuka Ikegame, Jun Shinoda

**Affiliations:** ^1^Department of Clinical Brain Sciences, Gifu University Graduate School of Medicine, Gifu, Gifu 501-1194, Japan; ^2^Chubu Medical Center for Prolonged Traumatic Brain Dysfunction, Kizawa Memorial Hospital, Minokamo, Gifu 505-0034, Japan

## Abstract

*Introduction.* Many patients with mild/moderate traumatic brain injury (m/mTBI) in the chronic stage suffer from executive brain function impairment. Analyzing brain metabolism is important for elucidating the pathological mechanisms associated with their symptoms. This study aimed to determine the differences in brain glucose metabolism between m/mTBI patients with and without visible traumatic brain lesions based on MRI.* Methods.* Ninety patients with chronic m/mTBI due to traffic accidents were enrolled and divided into two groups based on their MRI findings. Group A comprised 50 patients with visible lesions. Group B comprised 40 patients without visible lesions. Patients underwent FDG-PET scans following cognitive tests. FDG-PET images were analyzed using voxel-by-voxel univariate statistical tests.* Results.* There were no significant differences in the cognitive tests between Group A and Group B. Based on FDG-PET findings, brain metabolism significantly decreased in the orbital gyrus, cingulate gyrus, and medial thalamus but increased in the parietal and occipital convexity in Group A compared with that in the control. Compared with the control, patients in Group B exhibited no significant changes.* Conclusions.* These results suggest that different pathological mechanisms may underlie cognitive impairment in m/mTBI patients with and without organic brain damage.

## 1. Introduction

Executive brain function impairment, including cognitive, behavioral, and emotional impairment, may be observed in patients during the chronic stage following traumatic brain injury (TBI) [1–3]. This impairment may result in severe social problems, which can hinder the participation of patients in domestic, social, and occupational settings. In the USA, 1.7 million people sustain TBIs of varying severity each year [4]. Mild TBI accounts for at least 75% of these injuries [5]. Following mild TBI, some patients experience a brief loss of consciousness. Other symptoms include headache, dizziness, memory loss, attention deficit, depression, and anxiety. Some of these conditions may persist for months or even years in as many as 30% of patients [4]. For moderate TBI, where patients consistently have more than a minimal loss of consciousness in the acute stage, permanent disability including executive brain function impairment is thought to occur in 66% of cases [4, 6]. Among these symptoms, executive brain function impairment may be overlooked in the chronic stage and the pathological mechanisms related to this impairment may be difficult to distinguish between TBI and disorders associated with trauma-related psychiatric conditions.

The use of T2^*∗*^-weighted imaging (T2^*∗*^WI), fluid-attenuated inversion recovery (FLAIR), and diffusion tensor imaging (DTI) enables the detection of minute traumatic brain lesions even during the chronic stage for patients with mild as well as moderate TBI [7, 8]. These advanced imaging modalities can determine whether the executive brain function impairment may be attributed to true traumatic brain lesions or not for each case. For decades, brain metabolic imaging, including ^99m^TC-ECD single photon emission computed tomography to evaluate cerebral blood flow and ^18^F-fluorodeoxyglucose positron emission tomography (FDG-PET) to evaluate cerebral glucose metabolism, has played important roles in elucidating the pathophysiology of TBI as well as non-TBI disorders, including Alzheimer's disease [9], dementia with Lewy bodies [9], mild cognitive impairment [9], frontotemporal lobe degeneration [9], and depression [10–12]. The symptoms of these non-TBI disorders are partly similar to those seen during the chronic stage in patients with TBI, but the distinct brain metabolism impairments found in these disorders have gradually been elucidated.

In patients with mild/moderate (m/m) TBI who have suspected executive brain function impairment after injury, some may have true traumatic brain lesions. Thus, analyses of altered brain metabolism may be important for elucidating the pathological mechanisms that underlie their impairments. We hypothesize that there may be differences in the impaired brain metabolic patterns of patients with and those without true traumatic brain lesions during the chronic stage.

The aim of this study was to compare brain glucose metabolism in chronic m/mTBI patients with and without visible brain lesions based on MRI using FDG-PET.

## 2. Methods

### 2.1. Patients

In total, two hundred forty-three consecutive patients with chronic (we defined the chronic phase as more than 6 months after injury) m/mTBI due to traffic accidents were referred to the Kizawa Memorial Hospital between November, 2004, and May, 2010, because possible cognitive impairment was identified by the primary care hospital in the acute phase of the injury. In this study, we enrolled 90 patients excluding patients with large (≥3 mm) visible traumatic legions based on MRI (FLAIR), as well as those aged over 60 years and those under 20 years.

**Figure 1:**
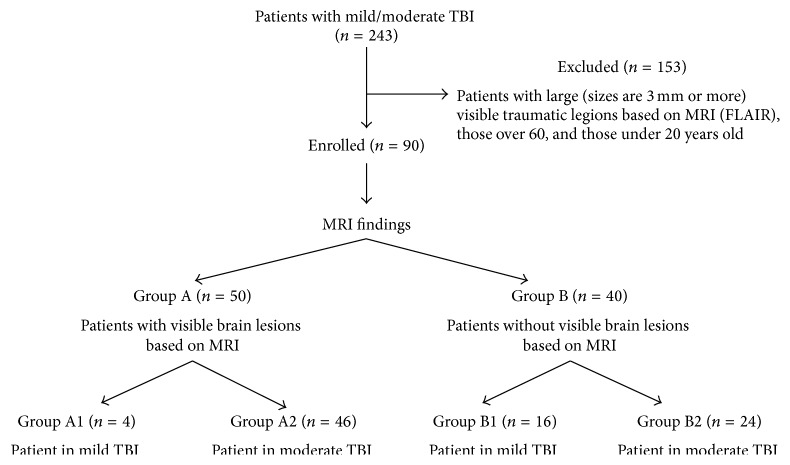
Flow chart illustrating the patient selection process.

According to the Glasgow Coma Scale (GCS) score evaluated in the primary care hospitals, patients were divided into mild TBI (GCS score = 13–15) or moderate TBI (GCS score = 9–12). Furthermore, the clinical criteria defined by the World Health Organization were employed for mild TBI [13]. No patient had a history of intracranial surgery or a penetrating head injury, open skull fracture, neurological disease unassociated with injury, mental retardation, psychiatric disease, or alcohol or substance abuse. The patients were diagnosed with diffuse brain injury (DBI) without massive contusional brain damage based on MRI performed more than six months after a traffic accident. The patients were divided into two groups (Groups A and B) according to their MRI findings with or without visible minute brain lesions (Figure 1). In this study, “visible minute brain lesions” were defined as lesions measuring <3 mm in diameter based on FLAIR, T2^*∗*^WI, and/or fractional anisotropy images analyzed by statistical parametric mapping (FA-SPM imaging) obtained from DTI [7].

Group A comprised 50 patients (mean age: 37.9 ± 16.9 years; 33 males) without any large traumatic lesions but with visible minute brain lesions based on MRI. The patients in Group A were further divided into two subgroups with mild TBI (Group A1: four patients) and moderate TBI (Group A2: 46 patients). Among the 50 patients in Group A, visible minute lesions with abnormally high signal intensity based on FLAIR were identified in the medial frontobasal regions, corpus callosum, and/or cingulate gyrus (21 patients); those with an abnormally low signal intensity based on T2^*∗*^WI were identified in the corpus callosum and/or subcortical regions of the cerebral hemisphere; and those with significantly decreased FA values in FA-SPM imaging (40 patients) were identified in the deep white matter of the cerebral hemisphere, in the vicinity of the basal ganglia, and/or in the corpus callosum (39 patients).

Group B comprised 40 patients (mean age: 37.6 ± 11.2 years; 24 males) without any traumatic lesions, including visible brain lesions based on any of the MRI modalities. The patients in Group B were further divided into two subgroups with mild TBI (Group B1: 16 patients) and moderate TBI (Group B2: 24 patients).

After the MRI scan, patients underwent FDG-PET scans and subsequent cognitive testing within two weeks, which included Hasegawa's Dementia Scale Revised (HDS-R), Mini-Mental State Examination (MMSE), Wechsler Intelligent Scale Revised (WAIS-R), and Wechsler Memory Scale Revised (WMS-R). As the control group, 75 healthy adult volunteers (mean age: 34.7 ± 15.3 years) also underwent FDG-PET scans. The interval between injury and all scans was at least 6 months in this study for all of the patients (average 36.2 ± 37.9 months). All of the patients had graduated from high school at least.

### 2.2. MRI

Whole-brain conventional MR imaging and DTI were acquired with a 1.5-Tesla MRI scanner (GE Medical Systems, Milwaukee, WI) using a quadrature transmit-receive head coil. The conventional MR imaging protocol included T2-weighted fast-spin echo (repetition time (TR)/echo time (TE)/number of excitations (NEX) = 2600 ms/107 ms/20), T1-weighted FLAIR (TR/TE/inversion time/NEX = 1850 ms/34 ms/805 ms/1), T2-weighted FLAIR (TR/TE/inversion time/NEX = 8000 ms/115 ms/2000 ms/1), and T2^*∗*^ GRE sequence (TR/TE/NEX/flip angle = 1550 ms/18 ms/1/20). In all of these sequences, a total of 16 contiguous sections were acquired in the axial plane with a slice thickness of 6 mm, matrix size of 512 × 256, and a field of view (FOV) measuring 240 mm × 240 mm. DTI was acquired with a single-shot echo planar sequence (TR/TE/NEX = 10000 ms/80 ms/4, slice thickness = 3 mm, matrix size = 128 × 128, and FOV = 250 × 250). Diffusion gradients were set in six noncollinear directions using two *b* values (*b* = 0 and 1000 s/mm^2^).

### 2.3. Date Analysis of FA Maps

Individual FA maps were calculated using an Advantage Workstation (GE Medical Systems). The structural distortion induced by large diffusion gradients was corrected based on T2-weighted echo planar images (*b* = 0 s/mm^2^). The FA maps were analyzed using statistical parametric mapping software (SPM8; Wellcome Department of Imaging Neuroscience, London, UK) in MATLAB version 6.5.1 (Mathworks, Natick, Massachusetts, USA).

First, all of the images were spatially normalized against a standard template. *b* = 0 images, which were T2-weighted images obtained without a diffusion gradient, were normalized against the standardized T2 template using linear transformations.

The FA maps were then normalized using the parameters determined from the normalization of *b* = 0 images. The normalized FA maps were smoothed with an 8 mm full-width at half-maximum isotropic Gaussian kernel to improve the signal-to-noise ratio, which increased the validity of the statistical inference and improved the normalization. An absolute masking with a threshold of 0.15 was used to exclude voxels outside the brain or in the ventricles from the analysis.

After the images had been spatially normalized and smoothed, the images of patients were compared statistically with those obtained from the controls in a voxel-wise manner using SPM8. In these analyses, a two-sample *t*-test was performed for each patient, so each patient was compared as one group (*n* = 1) with the age-matched control group. The SMP for both the between-group analysis and the individual-to-group analysis was thresholded to an uncorrected *p* value < 0.001 at the voxel level and to a minimum cluster size of 10 voxels. Two contrasts were used to detect whether each voxel had higher or lower anisotropy in both the between-group analysis and the individual-to-group analysis. The SPM, that is, the FA-SPM image, was projected onto a glass brain orthogonal projection.

### 2.4. FDG-PET

The PET scanner used in this study was an Advanced NXi imaging system (General Electric Yokohama Medical Systems, Tokyo, Japan), which provides 35 transaxial slices at 4.24 mm intervals. The in-plane spatial resolution (full-width at half-maximum) was 4.8 mm. The scans were acquired in the standard two-dimensional mode. Participants were placed in the PET scanner so the slices were parallel to the canthomeatal line. Immobility was checked by the alignment of three laser beams and lines drawn on the participants' faces. Participants fasted for at least 4 h prior to the injection of FDG. A dose of 0.12 mCi/kg of FDG was injected intravenously over a period of 1 min. After resting for 40 min, 3-min transmission scans were obtained using a ^68^Ge/^68^Ga rotating pin source. A static PET scan was performed continuously for 7 min in the awake and resting states. The static scan was reconstructed by correcting for photon attenuation using the data obtained from the transmission scans, dead time, random, and scatter [14].

### 2.5. Date Analysis of FDG-PET

FDG-PET images were processed and analyzed on a Microsoft workstation with MATLAB software (Mathworks, Sherborn, Massachusetts, USA) and SPM8 software (courtesy of the Functional Imaging Laboratory, Wellcome Department of Cognitive Neurology, University College London, London, UK).

First, the images were subjected to spatial transformations so they matched a template that conformed to the space derived from standard brains from the Montreal Neurological Institute (https://www.bic.mni.mcgill.ca/). These images were then smoothed with a Gaussian low-pass filter of 8 mm in-plane to minimize noise and improve the between-subject spatial alignment. After smoothing, an appropriate linear statistical model was applied to each voxel. Finally, statistical inference was employed to correct for the multiple dependent comparison using the theory of Gaussian fields.

The images were spatially normalized and a smoothed, general linear model [15, 16] was used to conduct appropriate voxel-by-voxel univariate statistical tests in the form of group comparisons (two-sample *t*-tests and regression analysis) between m/mTBI groups and normal controls. The differences in image intensity between participants were normalized to prevent interparticipant variability in cerebral tracer uptake from masking regional changes. This process was performed using the technique of proportional scaling [17], which scales each image according to a reference count, the global brain activity, or counts in a specific region. Therefore, these image data were scaled in proportion to the mean global brain activity [18]. The gray matter threshold was set to 0.8 because this appeared to effectively segment the brain regions from tissues outside the brain as well as from the ventricles. Brain regions with an uncorrected *p* value < 0.001 and FWE corrected *p* value < 0.05 for multiple comparisons of random field theory [19–21] were investigated for clusters with a minimum of 100 contiguous voxels. To visualize the *t*-score statistics (SPM *t*-map), significant voxels were projected onto the glass brain in three orthogonal projections provided by SPM8.

## 3. Results

According to the regression analysis of Group A, general memory, verbal memory, and delayed memory based on WMS-R were correlated significantly with hypometabolism based on FDG-PET. General memory was correlated significantly in the right thalamus, verbal memory was correlated in the precentral gyrus, the inferior frontal gyrus, and the insula of the right hemisphere, and delayed memory was correlated in the medial frontal gyrus of the right hemisphere. In Group B, there were no significant correlations between FDG uptake and cognitive function.

The results of the cognitive tests are shown for the patients in Tables 1, 2, and 3. There were no significant differences in the cognitive tests between all patients with and without visible minute traumatic lesions (two-sample *t*-test at *p* < 0.05) (Tables 1, 2, and 3).

**Figure 2:**
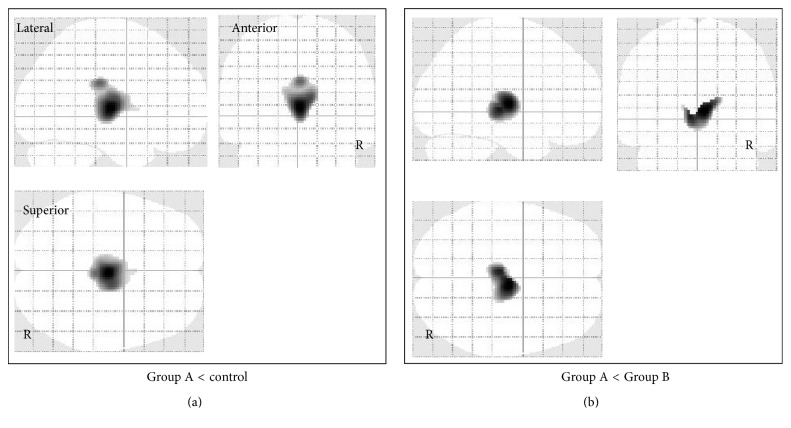
Voxel-based analysis of glucose metabolism data obtained by FDG-PET imaging in all patients with mild/moderate TBI. SPM in orthogonal maximum-intensity projections showing voxels with significantly lower FDG uptake (shaded areas, FWE-corrected *p* < 0.05) in Group A versus control group (a) and in Group A versus Group B (b). The shading intensity shows the overlap of hypometabolic regions from three directions (lateral, anterior, and superior). Brain metabolism was significantly lower in the cingulate gyrus and medial thalamus in Group A compared with the control group and lower in the medial thalamus in Group A compared with Group B.

**Figure 3:**
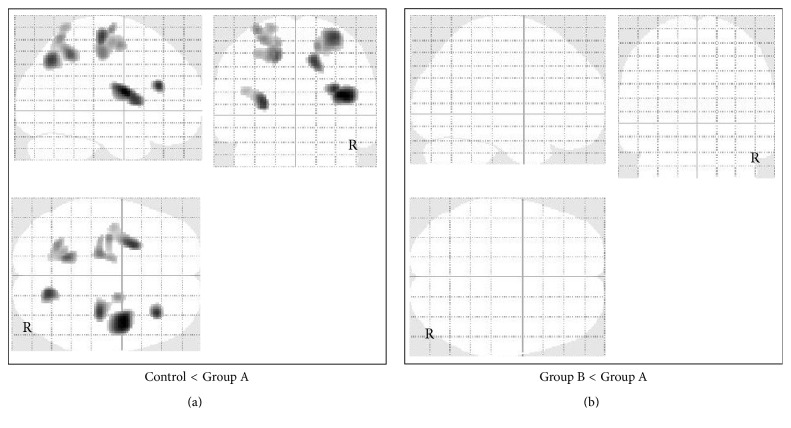
Voxel-based analysis of glucose metabolism data obtained by FDG-PET imaging in all patients with mild/moderate TBI. SPM in orthogonal maximum-intensity projections showing the voxels with significantly higher FDG uptake (shaded areas, FWE-corrected *p* < 0.05) in Group A versus control group (a) and in Group A versus Group B (b). The shading intensity shows the overlap of hypermetabolic regions from three directions (lateral, anterior, and superior). Brain metabolism was significantly higher in the parietal and occipital convexity in Group A compared with the control group, but there was no significant hypermetabolism compared with Group B.

According to FDG-PET, FDG uptake was significantly lower in the cingulate gyrus and medial thalamus in Group A (50 patients) compared with Group B (40 patients) and lower in the medial thalamus compared with the control group (Figure 2). Significantly higher FDG uptake was detected in the parietal and occipital convexity in Group A compared with the control group, but there was no significant increase in FDG uptake in Group A compared with Group B (Figure 3). In patients from Group B, there were no significant changes in FDG uptake compared with the control group.

In patients with mild TBI, there were no significant differences in FDG uptake among Groups A1 (4 patients) and B1 (16 patients) and the control group.

**Figure 4:**
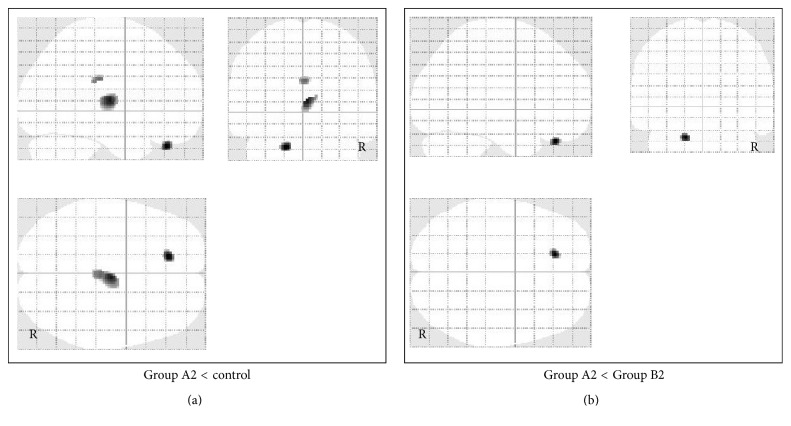
Voxel-based analysis of glucose metabolism data obtained by FDG-PET imaging in patients with moderate TBI. SPM in orthogonal maximum-intensity projections showing the voxels with significantly lower FDG uptake (shaded areas, FWE-corrected *p* < 0.05) in Group A2 versus control group (a) and in Group A2 versus Group B2 (b). The shading intensity shows the overlap of hypometabolic regions from three directions (lateral, anterior, and superior). Brain metabolism was significantly lower in the left orbital gyrus, cingulate gyrus, and right thalamus in Group A2 compared with the control group and lower in the left orbital gyrus compared with Group B2.

**Figure 5:**
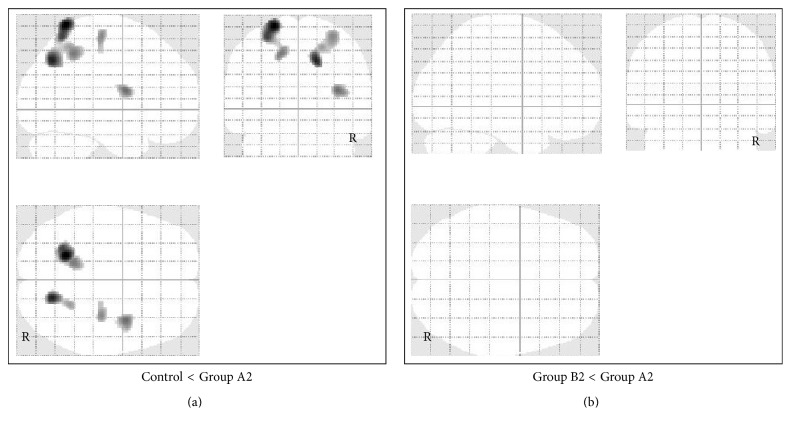
Voxel-based analysis of glucose metabolism data obtained by FDG-PET imaging in patients with moderate TBI. SPM in orthogonal maximum-intensity projections showing voxels with significantly higher FDG uptake (shaded areas, FWE-corrected *p* < 0.05) in Group A2 versus control group (a) and in Group A2 versus Group B2 (b). The shading intensity shows the overlap of hypermetabolic regions from three directions (lateral, anterior, and superior). Brain metabolism was significantly higher in the parietal and occipital convexity in Group A2 compared with the control group, but there was no significant hypermetabolism compared with Group B2.

In patients with moderate TBI, FDG uptake was significantly lower in the left orbital gyrus, cingulate gyrus, and right thalamus in Group A2 (46 patients) compared with the control group and significantly lower in the left orbital gyrus compared with Group B2 (24 patients) (Figure 4). Significantly higher FDG uptake was detected in the parietal and occipital convexity in Group A2 compared with the control group, but no significant increase in FDG uptake was found in Group A2 compared to Group B2 (Figure 5).

## 4. Discussion

The pathological mechanisms that underlie executive brain function impairments, including cognitive, behavioral, and emotional impairments, have yet to be elucidated in patients during the chronic stage following TBI. Representative symptoms of these impairments are memory disturbance, decreased activity, failure of emotional control, irascibility, carelessness, excessive tenacity, planning and execution failure, and excessive dependence. Indeed, diverse pathologies may underlie these symptoms. In the vast majority of severe TBI cases, substantial morphological brain lesions due to TBI can be observed by neuroimaging and the pathological mechanisms related to impaired executive brain function can be easily understood. However, it may be more difficult to identify the underlying pathological mechanisms in cases of m/mTBI where morphological brain lesions cannot necessarily be distinguished by neuroimaging. The symptoms may be due to organic pathologies (true traumatic lesions due to TBI) in some cases, trauma-related psychiatric conditions in others, or both.

The clinical application of DTI for investigating organic brain damage after TBI has detected significantly reduced brain integrity mainly in the corpus callosum and fornix in patients without macroscopic lesions after DBI [18]. Some patients with m/mTBI during the chronic stage have been reported to exhibit abnormally reduced white matter integrity in certain brain regions [22]. These brain regions were strongly associated with chronic cognitive impairment according to tract-based spatial statistics analysis using DTI [22].

Examination of cerebral metabolism in patients with TBI during the chronic stage using FDG-PET may allow us to elucidate the pathological mechanisms that underlie executive brain function impairment. In a previous FDG-PET study, hypometabolism was consistently indicated bilaterally in the medial prefrontal regions, the medial frontobasal regions, the cingulate gyrus, and the thalamus in patients with vegetative and minimally conscious states and those with executive brain function impairment after DBI [23]. This pattern of hypometabolism, which may reflect clinical deterioration, could be due to functional and structural disconnections of neural networks but not necessarily due to direct focal morphological damage de novo because inconsistency between regions of hypometabolism has been reported based on FDG-PET and reduced integrity based on DTI [18, 23].

In this study, brain metabolism was significantly lower in the anterior basal regions, cingulate gyrus, and medial thalamus of patients with visible minute brain lesions based on MRI (Group A) compared with the control group. These results are consistent with those observed in patients after DBI reported in the previous study mentioned above [24–26]. However, significant hypermetabolism was observed in the parietal and occipital convexity compared with the control group. This finding suggests that metabolism in the parietal and occipital convexity was enhanced to compensate for hypometabolism in the anterior basal regions, cingulate gyrus, and medial thalamus in Group A. Interestingly, some reports [27, 28] have shown that brain metabolic changes can be seen even in mild TBI patients without visible lesions, which contradicts our results. This difference may be explained by the actual inclusion of patients with visible lesions among the TBI patients without visible lesions enrolled in previous studies. Our study included DTI with FA-SPM analysis to detect lesions, which may be more sensitive for detecting minute lesions compared with conventional CT/MRI.

According to FDG-PET of patients with depression, reduced glucose metabolism in the dorsolateral and medial regions of the bilateral anterior and posterior frontal areas is the most consistent finding [10, 12]. By contrast, a characteristic increase in glucose metabolism was observed in the subgenual and pregenual anterior cingulate cortices in these patients [11]. The mechanism of trauma-related psychiatric conditions is considered to be somewhat similar to that of depression. Patients with such trauma-related psychiatric conditions might have been included in Group B, which may partly explain the difference in the glucose hypometabolic pattern between Group A and Group B.

Subanalytically there were no significant changes in glucose metabolism between Group A1 and Group B1, but there were significant metabolic changes between Group A2 and Group B2. This difference may suggest that the severity of TBI during the acute phase influenced glucose metabolism in the chronic phase.

These results of this study suggest that different pathological mechanisms related to cognitive impairments may exist in patients with chronic m/mTBI with and without organic brain damage. Another possible interpretation of the results is that the identification of hypometabolic regions based on FDG-PET may distinguish symptoms induced by executive brain function impairment due to organic brain damage after TBI from those due to functional pathologies in trauma-related psychiatric conditions.

## 5. Conclusions

This FDG-PET study showed that brain metabolism was significantly lower in the medial prefrontal regions, cingulate gyrus, and medial thalamus but significantly higher in the parietal and occipital convexity in patients with chronic m/mTBI and visible brain lesions based on MRI compared with patients without visible brain lesions based on MRI as well as a healthy control group. These results suggest that different pathological mechanisms may underlie cognitive impairments in patients with chronic m/mTBI with and without organic brain damage.

## Figures and Tables

**Table 1:** Results of neuropsychological tests in patients with mild/moderate TBI.

Factor	Group A (*n* = 50)	Group B (*n* = 40)	*p*-value
HDS-R	24.5 ± 5.0	25.7 ± 4.3	0.260

MMSE	26.7 ± 3.6	27.4 ± 2.5	0.307

*WAIS-III*			
FIQ	80.1 ± 18.1	86.9 ± 18.6	0.103
VIQ	80.4 ± 16.2	87.4 ± 17.8	0.073
PIQ	83.0 ± 18.5	89.2 ± 19.2	0.148

*WMS-R*			
General memory	92.1 ± 16.6	94.6 ± 18.7	0.617
Verbal memory	93.5 ± 18.7	94.2 ± 18.0	0.884
Visual memory	91.3 ± 17.8	97.2 ± 15.2	0.192
Delayed memory	89.9 ± 19.6	94.6 ± 18.0	0.359
Attention	93.2 ± 13.8	89.2 ± 14.7	0.316

**Table 2:** Results of neuropsychological tests in patients with mild TBI.

Factor	Group A1 (*n* = 4)	Group B1 (*n* = 16)	*p*-value
HDS-R	24.8 ± 4.7	25.6 ± 4.5	0.719

MMSE	26.5 ± 3.9	27.6 ± 2.6	0.499

*WAIS-III*			
FIQ	85.7 ± 32.5	90.1 ± 18.1	0.722
VIQ	83.8 ± 24.3	91.0 ± 16.6	0.484
PIQ	82.8 ± 30.0	91.1 ± 20.0	0.504

*WMS-R*			
General memory	93.7 ± 13.6	95.1 ± 13.6	0.641
Verbal memory	93.3 ± 17.7	94.6 ± 13.7	0.699
Visual memory	97.0 ± 5.0	95.7 ± 11.1	0.681
Delayed memory	96.7 ± 13.4	91.3 ± 16.0	0.930
Attention	100.3 ± 18.0	93.0 ± 9.8	0.210

**Table 3:** Results of neuropsychological tests in patients with moderate TBI.

Factor	Group A2 (*n* = 46)	Group B2 (*n* = 24)	*p*-value
HDS-R	24.5 ± 5.1	25.0 ± 4.4	0.735

MMSE	26.7 ± 3.6	26.5 ± 3.0	0.845

*WAIS-III*			
FIQ	79.5 ± 15.9	82.5 ± 18.5	0.568
VIQ	80.3 ± 14.3	82.4 ± 18.2	0.648
PIQ	86.4 ± 21.7	86.6 ± 17.7	0.507

*WMS-R*			
General memory	91.9 ± 16.9	86.3 ± 27.6	0.504
Verbal memory	93.5 ± 18.8	87.3 ± 26.3	0.483
Visual memory	90.6 ± 18.7	90.7 ± 22.6	0.985
Delayed memory	89.0 ± 20.1	86.7 ± 21.5	0.811
Attention	92.3 ± 12.9	85.0 ± 23.8	0.283

## Abbreviations

FA: Fractional anisotropy
FDG-PET: ^18^F-fluorodeoxyglucose positron emission tomography
PTSD: Posttraumatic stress disorder
SPM: Statistical parametric mapping
TBI: Traumatic brain injury.
