# Corrigendum to “Differences in Brain Metabolic Impairment between Chronic Mild/Moderate TBI Patients with and without Visible Brain Lesions Based on MRI”

**DOI:** 10.1155/2017/6019403

**Published:** 2017-05-30

**Authors:** Keiichi Ito, Yoshitaka Asano, Yuka Ikegame, Jun Shinoda

**Affiliations:** ^1^Department of Clinical Brain Sciences, Gifu University Graduate School of Medicine, Gifu, Gifu 501-1194, Japan; ^2^Chubu Medical Center for Prolonged Traumatic Brain Dysfunction, Kizawa Memorial Hospital, Minokamo, Gifu 505-0034, Japan

In the article titled “Differences in Brain Metabolic Impairment between Chronic Mild/Moderate TBI Patients with and without Visible Brain Lesions Based on MRI” [1], there were errors in the following sections.

In the Abstract section, the sentence “There were no significant differences in the cognitive tests between Group A and Group B. Based on FDG-PET findings, brain metabolism significantly decreased in the orbital gyrus, cingulate gyrus, and medial thalamus but increased in the parietal and occipital convexity in Group A compared with that in the control” should be changed to “There were no significant differences in the cognitive tests between Group A and Group B. Based on FDG-PET findings, brain metabolism significantly decreased in the cingulate gyrus and medial thalamus but increased in the parietal and occipital convexity in Group A compared with that in the control.”

In the Results section, the sentence “According to FDG-PET, FDG uptake was significantly lower in the cingulate gyrus and medial thalamus in Group A (50 patients) compared with Group B (40 patients) and lower in the medial thalamus compared with the control group (Figure  2)” should be changed to “According to FDG-PET, FDG uptake was significantly lower in the cingulate gyrus and medial thalamus in Group A (50 patients) compared with the control group and lower in the medial thalamus compared with Group B (40 patients) (Figure  2).”

In the Conclusion section, the statement “This FDG-PET study showed that brain metabolism was significantly lower in the medial prefrontal regions, cingulate gyrus, and medial thalamus but significantly higher in the parietal and occipital convexity in patients with chronic m/mTBI and visible brain lesions based on MRI compared with patients without visible brain lesions based on MRI as well as a healthy control group” should be changed to “This FDG-PET study showed that brain metabolism was significantly lower in the cingulate gyrus and medial thalamus and significantly higher in the parietal and occipital convexity in patients with chronic m/mTBI with visible brain lesions based on MRI compared with a healthy control group, but there were no significant changes in FDG uptake in patients with chronic m/mTBI without visible brain lesions based on MRI compared with a healthy control group.”
